# Mirror neuron system

**DOI:** 10.4103/0019-5545.31522

**Published:** 2007

**Authors:** V. Rajmohan, E. Mohandas

**Affiliations:** Elite Mission Hospital, Thrissur, India

Mirror neuron system is a group of specialized neurons that “mirrors” the actions and behaviour of others. The involvement of mirror neuron system (MNS) is implicated in neurocognitive functions (social cognition, language, empathy, theory of mind) and neuropsychiatric disorders. MNS discovery is considered to be the most important landmark in neuroscience research during the last decade.

## MIRROR NEURON SYSTEM - DISCOVERY

The mirror neurons were discovered serendipitously by Giacomo Rizzolatti and colleagues while working on the grasp response of macaques. They observed that a group of neurons in the area F5 of the premotor cortex that fires when a macaque performs an action; also discharges when it observes the same action being performed by another animal.[1] Subsequent research has elucidated the diverse regions involved in the MNS of monkeys. Recently different cortical structures have been described as part of the MNS in humans.

### MNS in monkeys

The area F5 of the premotor cortex (premotor area located.in the posterior bank of the inferior arcuate sulcus and the cortical convexity immediately caudal to it) in monkeys has two sets of visuomotor neurons namely the ‘canonical’ and the ‘mirror neurons’. The ‘canonical neurons’ (in F5 bank region) respond to presentation of an object while mirror neurons (in F5 convexity) respond to performance of an action and observation of an object directed action.[2] The mirror neurons are triggered by any action that involves the interaction between a biological effector (mouth, hand etc.) and an object. They are stimulated by the observation of the exact same action involving the effector and object (‘strictly congruent’ neurons) and also by actions that are similar but not having exact effector-object interaction (‘broadly congruent’ neurons).[3]

**Figure 1:**
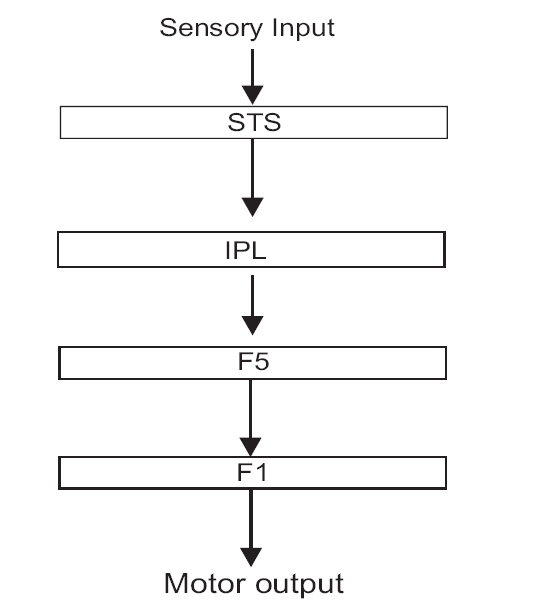
Basic mirror neuron circuitry in monkey. F1: Primary motor cortex, F5: Premotor cortex, IPL: inferior parietal lobule, STS: superior temporal sulcus.

Other areas that form part of the MNS are the superior temporal sulcus (STS) and the area 7b (PF of Von Economo) in the inferior parietal lobule (IPL). The STS codes for a larger number of movements than the F5 neurons but lacks motor properties (i.e., does not discharge while performing the movement). The area 7b neurons in IPL are heterogeneous and have a role in coding sensory stimuli and respond to somatosensory, visual or bimodal stimuli. I in addition to this, they also have motor properties and discharge on action observation and performance. The IPL receives inputs from the STS and sends an important output to the ventral premotor cortex including area F5 [Figure 1].[3,4]

### MNS in humans

**Figure 2:**
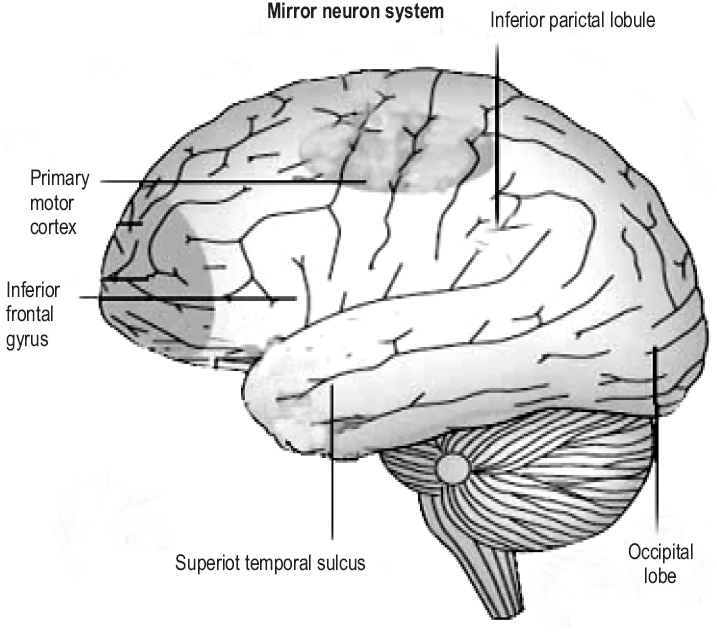
Mirror neuron regions in humans

Brain imaging studies reveal that action observation in humans activates the inferior frontal gyrus (IFG), lower part of the precentral gyrus, the rostral part of the IPL and also the temporal, occipital and parietal visual areas.[3] The frontal and the parietal mirror neuron regions are somatotopically organized. The activation of pars opercularis of the IFG reflects the observation of distal hand and mouth actions, whereas the activation of the premotor cortex reflects proximal arm and neck movements [Figure 2]. The mirror neurons in humans, unlike those in monkeys fire even while observing meaningless (intransitive) movements. The observation of transitive actions causes the firing of the frontal and the temporal nodes of the MNS while that of intransitive actions result in the firing of the frontal node only.[5,6]

## FUNCTIONS OF MNS

### Action understanding

Action understanding is the fundamental function of the MNS. Each time the animal observes a certain action being performed by another animal, the mirror neurons representing the performance of that action are activated. The mirror neurons transform visual observation into knowledge.[3] Studies on humans during action observation have shown activation of the IFG, the IPL and a region within the STS. The precentral motor cortex though not activated by action observation is involved indirectly in action understanding as they have a role in motor imagery.[7]

The main hypotheses to explain the phenomenon of action understanding are the visual hypothesis, direct-match hypothesis and the ‘generate and test’ model. Visual hypothesis is based on the visual analysis of the effector, the object and on the context in which the action is going on to draw conclusions as to the meaning of the action. The neural substrates are visual extrastriate areas, inferotemporal lobe and STS region. The direct match hypothesis is based on the mapping of observed action on his/her own motor representation of the observed action. Therefore it involves a process of observation induced motor representation, followed by matching of this to the motor representation generated during active outcome directed action performed by the individual (simulation). If both these motor representations correspond, it leads to action understanding.[4] A more complex hypothesis suggested for action understanding is the ‘generate and test’ model. According to this model, action understanding involves finding a ‘pretend’ goal that would generate an action plan in the observer's own motor planning system. This is then matched with the observed action. It is presumed that when the simulated motor action does not match the observed one, a new hypothesis is generated and tested for congruence with the observed action. So actions are understood not just in terms of their outcomes, but also in terms of the mental states and especially the goals, that have generated them.[8]

### Imitation

Basic circuit underlying imitation coincides with that which is active during action observation. Imitation requires a perfect matching of the performed action onto the observed one. Mirror neurons are able to recognize the actions of others and the intention associated with them. So they can code for likely future actions of others, thereby observers are able to anticipate the actions of others.[1,9]

Human imitation involves flow of information through the STS, IPL and the IFG. STS provides higher order visual processing of observed action while the fronto- parietal MNS codes for the goal of the action and the motor plan on how to achieve it. The fronto- parietal system then sends copies of this motor plan to the STS, which matches the predicted sensory consequence of the planned motor action with the visual description of the observed action. The efferent copies to the STS originate from the ventral sector of the pars opercularis of the IFG, the activity of which is specific to imitation.[9]

**Figure 3:**
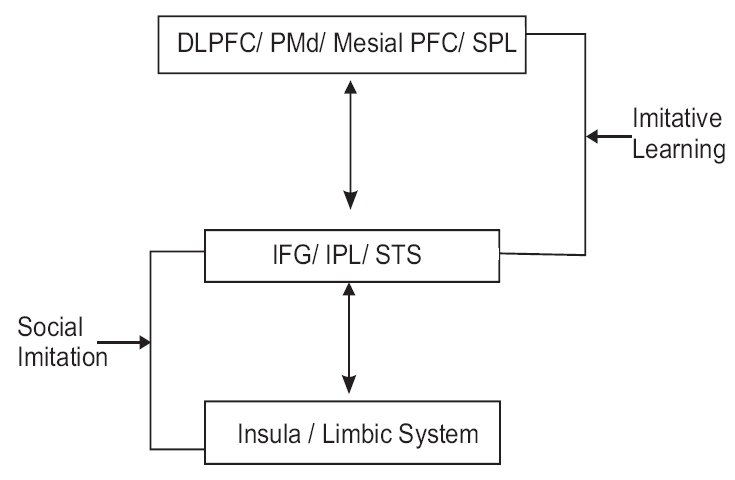
Mirror neuron structures in imitation

There is a difference during imitative learning of tasks between novel tasks and the tasks that are part of the observer's motor repertoire. The frontal and parietal areas are activated during observation of a task that is part of one's repertoire. The imitation of novel action in addition to the above regions involves the dorsolateral prefrontal cortex (DLPFC) and the cortical areas relevant to motor preparation namely- dorsal premotor cortex (PMd), mesial prefrontal cortex and the superior parietal lobule. So the basic imitation learning circuit consists of IFG, IPL, STS, motor preparation areas and DLPFC [Figure 3].[9]

### Speech and language

The presence of mirror neurons in Broca's area of humans suggests that human language may have evolved from a gesture performance/understanding system. The tasks like spontaneous speech and reading activate the hand motor area and the IFG, on the left side.[10,11] So language mirror neurons seem to be lateralized to the left side involving the dominant hand motor cortex and the higher levels of language network.[12]

### Theory of mind

Theory of mind (ToM) or mentalisation is the ability to recognize that someone else has a mind different from one's own. It involves the ability to infer someone else's mind by facial expression, tone of voice and non-verbal communication. It involves the area concerned with action imitation, face imitation and intention understanding. The neural structures involved in ToM include IFG, right STS, right IPL, medial prefrontal cortex including the anterior cingulate cortex (ACC) orbitofrontal cortex (OFC), precuneus, somatosensory cortex, amygdala and the occipital cortex. Therefore the MNS is considered integral to the theory of mind.[13,14]

### Social communication and empathy

Social communication and identification involve imitation. The more people tend to imitate each other, the more they are able to develop an empathic relationship. Social mirroring involves the interaction of the core mirror neuron system with the limbic system. Imaging studies have shown that observation and imitation of facial emotional expression involve the fronto-parietal mirror neuron system, STS, insula and the limbic system [Figure 3].[9]

Empathy is a process which involves the affective sharing between self and others, adopting the perspective of others and the ability for self agency and self regulation. The neural correlates empathy include IFG, right STS, right IPL, ACC, ventromedial prefrontal cortex (VMPFC), somatosensory cortex, amygdala, precuneus, insula and the posterior cingulate. Thus empathy involves a significant interaction of the core MNS and its limbic extension[9] [Table 1].

**Table 1:** Neural correlates of mirror neuron system, theory of mind and empathy

Mirror system	Theory of mind	Empathy
IFG	IFG	Premotor (IFG)
STS	STS (Rt)	STS (Rt)
IPL (Rt)	IPL (Rt)	IPL (Rt)
Occipital	Occipital	ACC
Primary motor cortex	Med. PFC (inc ACC) OFC Precuneus Somatosensory cortex Amygdala	VMPFC (inc OFC) Precuneus Somatosensory cortex Amygdala Insula Posterior cingulate

IFG - Inferior frontal gyrus, STS - Superior temporal sulcus, IPL- Inferior parietal lobule, PFC - Prefrontal cortex, OFC - Orbitofrontal cortex, ACC - Anterior cingulate cortex

### Social cognition

Social cognition refers to thought processes involved in understanding and dealing with others. It involves regions that mediate face perception, emotional processing, theory of mind, empathy (especially self reference) and working memory. The MNS involvement in mediating empathy and ToM reflects its significant role in social cognition.[15]

### Mirror neurons and neuropsychiatric disorders

Autism and autism spectrum disorders (ASD) are characterized by social impairment, the lack of ToM and demonstrable defects in imitation skills. This implies dysfunctional MNS in ASD. Neuroimaging studies have demonstrated lesser activation of Broadman's area (BA) 44/45, the superior temporal gyrus (BA 22), the right insula and the left amygdala.[16] A recent study also demonstrated the lack of MNS activity during observation and emotional expression in children with ASD.[17]

The mirror neurons may also be involved in the development of disorders with hypersociality like William's syndrome and Turner's syndrome. The relevance of mirror neurons in social cognition may account for dysfunctional MNS in social phobia and asociality observed in schizophrenia.[15]

The MNS also plays an important role in bonding and attachment. Dysfunctional MNS may play a role in antisocial and borderline personality disorder. The patients with borderline personality disorder lack the ability to discern the mental states of self and others. They have fractured early attachments leading to a deficiency in learning of the concepts like secure attachment and the capacity to mentalise.[9,13] Psychotherapeutic processes involve the MNS accounting for empathy and ToM.[13]

## CONCLUSION

The fascinating discovery of MNS has generated tremendous enthusiasm among researchers in cognitive neuroscience. The involvement of MNS in social cognition, empathy and ToM has fuelled interest in explaining its role in ASD, schizophrenia, personality disorders and psychotherapeutic processes. The future research may unravel mysteries surrounding MNS.
